# Editorial: Next generation staging in head and neck cancers

**DOI:** 10.3389/fonc.2022.1106125

**Published:** 2022-12-14

**Authors:** N. Gopalakrishna Iyer, Narayana Subramaniam, Cesare Piazza

**Affiliations:** ^1^ Department of Head and Neck Surgery, Singapore General Hospital and National Cancer Centre, Singapore and Duke National University of Singapore (NUS) Medical School, Singapore, Singapore; ^2^ Head and Neck Oncology, Sri Shankara Cancer Hospital and Research Centre, Bengaluru, India; ^3^ Unit of Otorhinolaryngology – Head and Neck Surgery, ASST Spedali Civili of Brescia, Department of Surgical and Medical Specialties, Radiological Sciences and Public Health, University of Brescia, School of Medicine, Brescia, Italy

**Keywords:** staging, radiomics, biomarkers, molecular markers, prognosis, squamous cell carcinoma, head and neck cancer

Despite numerous advances in treatment, head and neck squamous cell carcinoma (HNSCC) has remained an important cause of cancer-related morbidity and mortality the world over. Until 2017, when the 8^th^ Edition of the TNM staging system of the Union for International Cancer Control/American Joint Committee on Cancer was released, staging had remained relatively unchanged for decades. The new staging system acknowledged the role of depth of invasion in oral cancer, as well as prognostic determinants in HPV-positive oropharyngeal tumors and virus-related (including HPV and EBV-associated) unknown primaries (1), representing its intention to evolve from a population-based staging system to a more ‘personalized’ approach. Even with these improvements, conventional TNM staging has been found to have drawbacks difficult to be addressed (2). While TNM staging is focused on prediction of overall survival using well-established, historical criteria, there have been constant attempts at improving it by incorporating pathologic, radiologic, genomic, and other biomarkers. This serves several purposes: to improve precision and accuracy in existing staging systems, to sub-stratify patients within a stage to predict survival more accurately, and to identify those who are candidates for treatment escalation through clinical trials and other novel therapeutic strategies.

In the quest to improve the present staging system, there is an almost reflexive instinct to improve it by increasing the number of parameters included and, thus, the inherent complexity of the overall process, but this approach is not without drawbacks. In fact, it results in increased heterogeneity of staging, across geographies and institutions, which are difficult to account for (3). As the complexity and number of parameters included increase, there is inevitably also a parallel reduction of the inter-observer agreement. This, in turn, impacts the overall reliability of the staging system itself. Herein lies the challenge: how do we improve staging methodology to reflect our better understanding of the disease, while maintaining its simplicity, applicability, and reproducibility? The answer to this question, we believe, is probably nuanced. As our knowledge and understanding of the tumor behavior and biology improve, the TNM staging needs to reflect this; however, staging, by nature, needs to be simple, easy to understand and apply, and standardized across the world. As we consider tumor-related as well as patient-related factors that better prognosticate survival, it becomes important to understand the context in which they need to be applied. Parameters with a strong prognostic relevance which are universally applicable features, easy to be interpreted with little to no inter-observer variability are suitable for incorporation into staging, while others are likely to be better suited for a nomogram or a decision-making tool, which may help guide treatment decisions.

Next-generation HNSCC staging represents the understanding of the role that novel markers are likely to play in the diagnosis and treatment of such a dismal disease. Patient-specific models allow incorporating these factors into prognostication and treatment planning; in other cancers, like breast and prostate, these have become a common part of practice, while in HNSCC they have not yet become widely accepted. In the era of personalized medicine, this is even more relevant. Studies which incorporate molecular or genomic data into therapeutic decisions are unlikely to accrue the same number of patients as a traditional phase III clinical trial due to financial and other logistical hurdles, however the value they add cannot be discounted. It is important to be able to incorporate this newer data, as it appears, into practice to improve the quality of decision making, even if the number of patients studied is low. We are proud to present this issue, which is a good representation of such an expanding body of literature. The articles in this special issue cover novel clinical parameters, radiomics, and molecular markers that help predict survival, treatment response or improve prognostication, which continue to remain a pressing need in the treatment of patients with HNSCC.

## Author contributions

All authors listed have made a substantial, direct, and intellectual contribution to the work and approved it for publication.
